# Specific Oral Tolerance Induction Using IFN-Gamma in 2 Cases of Food-Dependent Exercise-Induced Anaphylaxis

**DOI:** 10.1155/2013/259692

**Published:** 2013-06-27

**Authors:** Geunwoong Noh, Jae Ho Lee

**Affiliations:** ^1^International Allergy Center, Pyeongtaek International Hospital, 109 Bangchuk-gil, Godeok-myeon, Pyeongtaek 451-841, Republic of Korea; ^2^Department of Paediatrics, Chungnam National University, Daejeon, Republic of Korea

## Abstract

Anaphylaxis induced by exercise after the intake of certain foods is referred to as food-dependent exercise-induced anaphylaxis (FDEIA). Only the preventive medication such as oral sodium cromoglycate and oral combined cetirizine-montelukast was tried in FDEIA. Specific oral tolerance induction (SOTI) using IFN-gamma was tried in 2 cases of FDEIA for wheat. Merely, exercise accompanied every treatment just after the intake of allergenic foods during treatment. Patients acquired tolerance for wheat in both cases successfully. After treatment, two patients take wheat in their food living freely. Conclusively, SOTI using IFN-gamma was effective as the causative treatment for allergenic foods in FDEIA.

## 1. Introduction

Anaphylaxis induced by exercise after the intake of certain foods is referred to as food-dependent exercise-induced anaphylaxis (FDEIA). Since Maulitz et al. [1] reported the first case of anaphylaxis induced by exercise after the intake of seafood in 1979, Kidd et al. [2] called this condition FDEIA with the report of a case of anaphylaxis after intake of celery in 1983. The most common causative food is wheat [3, 4]. For prevention of FDEIA, it is important not to eat the causative food before exercise and to avoid exercise after the intake of allergenic food [5].

There were several trials in FDEIA for the preventive medication such as oral sodium cromoglycate (SCG) [6] and oral combined cetirizine-montelukast [7]. However, there has been no causative treatment for FDEIA. Specific oral tolerance induction (SOTI) for food allergy has been tried recently [8]. In particular, SOTI using IFN-gamma was completely successful for IgE-mediated anaphylactic food allergy [9]. SOTI using IFN-gamma was successfully tried for FDEIA by wheat in this report.

## 2. Case Reports

### 2.1. Case 1

The patient was a 24-year-old woman weighing 44 kg. There was nothing notable in her family history. In 2008 (when she was 18 years old), she developed generalized urticaria, coughing, vomiting, diarrhea, pale complexion, and loss of consciousness with running after intake of pork cutlet for the first time. She visited the emergency room and was treated with emergency care for anaphylaxis. Thereafter, she showed several history of severe generalized urticaria after intake of foods which contain wheat such as pork cutlet, pizza, spaghetti, and noodle. However, she did not show anaphylactic reactions including systemic urticaria every time when she took food containing wheat. She had to visit the emergency room because of anaphylaxis two times in the similar situation. After that, wheat was eliminated in her food living and there have been no symptoms and signs. 

She visited the Republic of International Allergy Center, Pyeongtaek International Hospital (Pyeongtaek, Korea), for the tolerance induction for wheat from June 2012. It is recognized that anaphylaxis occurred with running until out of breath just after intake of food-containing foods in the past history. 

#### 2.1.1. Laboratory Tests

Blood eosinophil % was 0.3%. Serum total IgE level was 90.8 KU/L. Specific IgE levels were 1.13 KU/L for wheat and not detected for eggs, milk, and soybeans. However, specific IgE was 0.55 KU/L for Dp and 0.40 KU/L for Df. The results of skin prick test for wheat are +++ (wheal size were 4 mm for eggs, 4 mm for histamine, and 0 mm for normal saline). Skin prick test for eggs, milk, soybeans, Dp, and Df is negative.

#### 2.1.2. OFC

**Figure 1:**
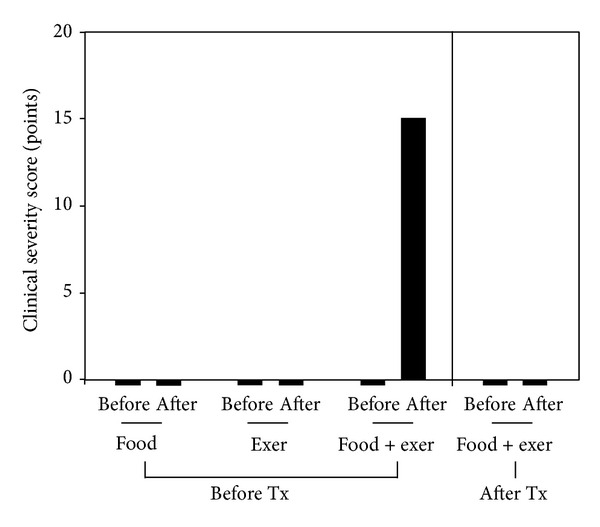
Diagnosis of food-dependent exercise-induced anaphylaxis (FDEIA) and the changes of clinical severity by oral challenge test and exercise provocation test (Case 1). Before: before challenge or exercise stimulation; after: after challenge or exercise provocation; food, oral challenge test for wheat; exer: exercise provocation test; food + exer: Exercise provocation just after intake of wheat; Tx: specific oral tolerance induction using IFN-gamma for wheat.

Exercise provocation without intake of wheat was performed by running until out of breath for 10 minutes. Patients did not show any symptoms and signs by exercise provocation test. Food challenge tests were conducted according to the protocol for IgE-mediated anaphylactic food allergy as described in the previous report [9]. Wheat was given 6 times for three days with amount of 120 g as breakfast and dinner. Also, patients did not show any symptoms and signs by oral challenge test. However, allergy provocation occurred with exercise just after exercise by running until out of breath for 10 minutes. The severity scores were 0 before and 2000 after oral food challenge with exercise at the dose of 10 g of noodle (Figure 1). The symptoms and signs were generalized urticaria with rash, respiratory difficulty with choking sensation, hypotension, and palpitation. The food-dependent, exercise-induced food allergy (FDEIA) for wheat was made.

#### 2.1.3. Treatment

Specific oral tolerance induction using IFN-gamma for wheat was performed. Noodle was used for wheat allergen. The patient took scheduled dose of wheat 10 minutes after receiving IFN-gamma 2,000,000 units for 10 minutes. After the intake of wheat, the patient ran for 10 minutes until out of breath. The initial dose is 0.01 g, and the dose of wheat was increased according to the protocol in the previous report [9]. Basically, the treatment followed the protocol for specific oral tolerance induction for IgE-mediated anaphylactic food allergy. The only difference from specific oral tolerance induction for food allergy was that the exercise was performed just after the intake of allergenic foods. 

**Figure 2:**
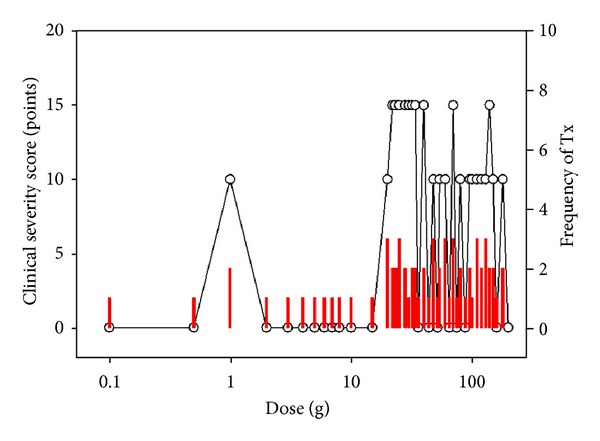
Clinical course of specific oral tolerance induction (SOTI) using IFN-gamma for food-dependent exercise-induced anaphylaxis (FDEIA) (Case 1). Solid line with spot is the change of clinical severity scores according to the dose. Bars are the frequency of treatment at the same dose to which patients showed allergy provocation by SOTI using IFN-gamma until overcoming the allergy provocation and resultant tolerance to the dose.

 The patient showed allergy provocation many times during the treatment at the variable doses (Figure 2). Emergency care was done and treatment was restarted after a week. The treatment was repeated for the same dose until allergy provocation was not occurred at the same dose according to the principle of overcome of the dose of allergy provocation. The treatment was finished after 4 months days with 90 times of treatment.

After treatment, patient the received oral food challenge with 120 g of wheat for the confirmation of tolerance acquisition. the patient show any symptoms and signs even with running just after intake of wheat no more for more than a year.

### 2.2. Case 2

The patient was a 36-year-old woman weighing 52 kg. There was nothing notable in her family history. In 2001 (when she was 27 years old), she developed generalized urticaria, dyspnea with chocking sensation of throat, vomiting, diarrhea, and loss of consciousness with running after intake of noodle for the first time. She also received emergency care for anaphylaxis. She showed frequent history of emergency care due to loss of consciousness as a symptom of anaphylaxis with running just after intake of wheat. Anaphylactic reactions never occurred only by wheat intake or only by exercise. She has kept restriction diet for wheat in her food living for the prevention of anaphylaxis. 

She visited the Republic of Allergy Center, Department of Paediatrics, Chungnam National University Hospital (Daejeon, Korea), for the tolerance induction for wheat in 2010. 

#### 2.2.1. Laboratory Tests

Blood eosinophil % was 5.1%. Serum total IgE level was 250.2 KU/L. Specific IgE levels were 4.1 KU/L for wheat and not detected for eggs, milk, and soybeans. However, specific IgE was 2.32 KU/L for Dp and 2.25 KU/L for Df. The results of skin prick test for wheat are +++ (wheal sizes were 3 mm for eggs, 3 mm for histamine and 0 mm for normal saline). Skin prick test for eggs, milk, soybeans, Dp, and Df is negative.

#### 2.2.2. OFC

She was negative in exercise provocation test without intake of wheat by running until out of breath for 10 minutes and in oral food challenge for wheat which was performed as described previously. Allergy provocation occurred at the dose of 30 g with exercise just after exercise by running until out of breath for 10 minutes with the severity scores of 2500. The symptoms and signs were generalized urticaria with rash, respiratory difficulty with choking sensation, hypotension, and palpitation. The food-dependent, exercise-induced food allergy (FDEIA) for wheat was made.

#### 2.2.3. Treatment

 SOTI using IFN-gamma was performed. Noodle was used for wheat allergen. Scheduled dose of wheat was tried 10 minutes after receiving IFN-gamma 2,000,000 units within 10 minutes. After the intake of wheat, she ran for 10 minutes until out of breath. The initial dose is 0.01 g, and the dose of wheat was increased according to the protocol. The treatment followed the protocol for specific oral tolerance induction for IgE-mediated anaphylactic food allergy with just addition of exercise just after the intake of allergenic food. 

The patient also showed allergy provocation many times during the treatment at the variable doses. Emergency care was done when she showed anaphylactic reactions. Treatment was restarted after a week. The treatment was repeated for the same dose until allergy provocation was not occurred at the same dose according to the principle of overcome of the dose of allergy provocation. The treatment was finished after 12 months days with 310 times of treatment.

 After treatment, patient received oral food challenge with 120 g of wheat for the confirmation of tolerance acquisition. The patient show any symptoms and signs even with running just after intake of wheat no more for more than 2 years.

## 3. Discussion

Specific oral tolerance induction using IFN-gamma was effective for the causative treatment of FDEIA. FDEIA is a form of IgE-mediated anaphylactic food allergy (IFA) caused by exercise after the intake of certain foods. The only difference between IFA and FDEIA is that FDEIA was provoked by exercise after intake of allergenic foods. Its major symptoms are also similar to IFA including generalized urticaria, angioedema (including facial swelling), respiratory impairment, hypotension, and disturbance of consciousness. Allergenic food in this report was wheat in both cases. The most common causative food was wheat, and it was reported that 60% of FDEIA was wheat dependent [3]. 

Elimination diet for the allergenic foods in FDEIA is a way of prevention [10]. For prevention of FDEIA, it is important to avoid exercise after causative food intake; see also [11]. However, patients possibly take foods containing allergenic foods without recognition because of many reasons. Preventive medication was also tried using oral sodium cromoglycate (SCG) [6] and oral combined cetirizine-montelukast [7]. When SCG was administered orally to food allergy patients before food intake, it reduced the symptoms. SOTI using IFN-gamma was completely successful in the treatment of IgE-mediated anaphylactic food allergy [9]. SOTI using IFN-gamma was applied to FDEIA and as we expected, SOTI using IFN-gamma was successful as a causative treatment with modified protocol in which exercise provocation should be included just after the intake of allergenic food. Both patients did not show any symptoms and signs with any exercise after intake of wheat for more than 2 years, and they got the tolerance for the allergenic foods. There has been no causative treatment for FDEIA except temporary preventive symptomatic treatment as described previously [6, 7]. However, the severity of disease is fatal, although the patients are always exposed to the causative allergens in their daily living. This treatment is recommended for all FDEIA patients.

 Allergy provocation and achievement of tolerance occurred in the low-dose zone for anaphylactic IgE-mediated food allergy and in the high-dose zone for non-IgE-mediated food allergy. Interestingly, the tolerance acquisition of tolerance occurred in the high-dose zone in FDEIA, even though the characteristics of FDEIA are IgE-mediated food allergy. IgE-mediated food allergy seems to be consisted of heterogeneous diseases.

Anaphylaxis is not the disease and rather a group of symptoms and signs which may be provoked by multiple causes. Moreover, exercise seems to be aggravation factor rather than the cause. So, exercise-induced food allergy (EIFA) may be a more precise terminology than food-dependent exercise-induced food allergy (FDEIA). Further clinical trial and basic studies may be needed. Conclusively, SOTI using IFN-gamma was effective as the causative treatment for allergenic foods in FDEIA or EIFA.

## Abbreviations

FDEIA: Food-dependent exercise-induced anaphylaxis
SOTI: Specific oral tolerance induction.
